# Recovery of Gallium-68 and Zinc from HNO_3_-Based Solution by Liquid–Liquid Extraction with Arylamino Phosphonates

**DOI:** 10.3390/molecules27238377

**Published:** 2022-12-01

**Authors:** Fedor Zhuravlev, Arif Gulzar, Lise Falborg

**Affiliations:** 1 Department of Health Technology, Section for Biotherapeutic Engineering and Drug Targeting, Technical University of Denmark, Frederiksborgvej 399, 4000 Roskilde, Denmark; 2 Regionshospitalet Gødstrup, Hospitalsparken 15, 7400 Herning, Denmark

**Keywords:** gallium-68, liquid–liquid extraction, liquid target, arylamino phosphonate, cyclotron production of gallium-68

## Abstract

The cyclotron production of gallium-68 via the ^68^Zn(*p,n*)^68^Ga nuclear reaction in liquid targets is gaining significant traction in clinics. This work describes (1) the synthesis of new arylamino phosphonates via the Kabachnik–Fields reaction, (2) their use for liquid–liquid extraction of ^68^Ga from 1 M Zn(NO_3_)_2_/0.01 M HNO_3_ in batch and continuous flow, and (3) the use of Raman spectroscopy as a process analytical technology (PAT) tool for in-line measurement of ^68^Zn. The highest extraction efficiencies were obtained with the extractants functionalized with trifluoromethyl substituents and ethylene glycol ponytails, which were able to extract up to 90% of gallium-68 in batch and 80% in flow. Only ppm amounts of zinc were co-extracted. The extraction efficiency was a function of pKa and the aqueous solubility of the extractant and showed marked concentration, solvent, and temperature dependence. Raman spectroscopy was found to be a promising PAT tool for the continuous production of gallium-68.

## 1. Introduction

Gallium-68 radiopharmaceuticals remain one of the cornerstones of positron emission tomography (PET). New ^68^Ga PET tracers significantly improve patients’ clinical outcomes, and the number of clinical trials and publications involving gallium-68 continues to grow. ^68^Ga-PSMA and ^68^Ga-NETSPOT™ (^68^Ga-SOMAkit-TOC) are becoming the gold standard for prostate cancer and neuroendocrine tumor diagnostics, [1] and Ga-FAPI is emerging as a new general PET tracer [2].

The clinical success of ^68^Ga radiotracers drives the soaring demand for ^68^Ga radionuclide. Most ^68^Ga is currently supplied by germanium-68 generators, which are convenient to use but expensive and in limited supply. The short life of germanium/gallium generators and their decreasing elution yield due to the decay of parent isotopes cause the total cost of generator ownership to be very high compared to the amount of ^68^Ga used for scans. An alternative technology that can potentially solve the ^68^Ga shortage has emerged. It is based on irradiation of the stable ^68^Zn isotope using medical cyclotrons. The ^68^Zn(*p,n*)^68^Ga nuclear reaction has a large cross-section and can provide ^68^Ga with high radionuclidic purity [3]. Traditionally, proton bombardment of solid targets has been used as a primary method of producing PET radiometals. The production of multi-curie amounts of ^68^Ga has been reported by using electrodeposited [4], pressed [5], and fused [6,7] ^68^Zn solid targets. However, this requires specialized and costly equipment for target preparation, irradiation, cooling, transportation, and post-irradiation target dissolution [8]. In 2011, Jensen and Clark showed that irradiation of a ^18^F liquid target charged with a concentrated solution of ^68^ZnCl_2_ can produce clinically-relevant yields of ^68^Ga [9]. It was subsequently recognized that liquid targets have a number of advantages over solid targets. Most importantly, liquid target-based production could be readily deployed within the existing clinical infrastructure designed and built for ^18^F radiochemistry. The production workflow benefited from easy target preparation, as well as from fast and automated delivery of the irradiated target solution to the hot cells for recovery and purification of ^68^Ga. All of this could be performed using the standard ^18^F targets and automated radiosynthesis modules. The initial challenges associated with significant outgassing during proton bombardment were overcome by the use of nitric acid solutions of metal nitrate salts [10]. Such solutions are commercially available from Fluidomica (www.fluidomica.pt; accessed on 29 November 2022). The Pandey [11,12,13] and Alves [14,15,16] research groups, as well as others [17,18], reported consistent and reliable production of ^68^Ga by irradiating liquid targets containing a zinc-68 nitrate solution made by dissolving ^68^Zn(NO_3_)_2_ (0.6–1.7 M) in nitric acid (0.01–0.3 M) with 30–45 µA proton beams for 30–60 min, yielding as much as 5 GBq at the end of the bombardment. Large regional clinics such as the Mayo Clinic (US) and ICNAS (Portugal) now routinely produce several batches of ^68^Ga per day using liquid targets. The European Pharmacopoeia monograph, which regulates the quality of cyclotron-produced ^68^Ga, has recently become available [19].

All current solid and liquid target ^68^Ga production methods use two-column solid phase extraction (SPE) as the means of purification. The first column, loaded with either hydroxamate [12,18,20] or a strong cation resin [4,5,15] (Dowex 50W-X8, AG 50W-X8), takes advantage of the stronger Lewis acidity of Ga^3+^ retained on the column, while Zn^2+^ is eluted with a HCl, HNO_3_, or acetone/HBr mixture. The second column, which may contain a phosphine oxide (TK200) [18,20], a cation exchange (AG1) [13,15], or a phosphonate (UTEVA) [4,5] resin, serves mainly to concentrate activity for elution in a formulation-friendly media, such as water or 0.1 M HCl. Since the cost of ^68^Zn has a relatively high impact on the overall price of cyclotron-produced ^68^Ga, reuse of ^68^Zn is desirable. The main limitation of the SPE methodology, at least at the current level of development, is that ^68^Zn cannot be immediately reused after SPE purification. The sorption on the first column and the subsequent elution changes both the zinc-68 concentration and the chemical composition of the solution, making it unsuitable for direct use in the cyclotron liquid target.

**Figure 1:**
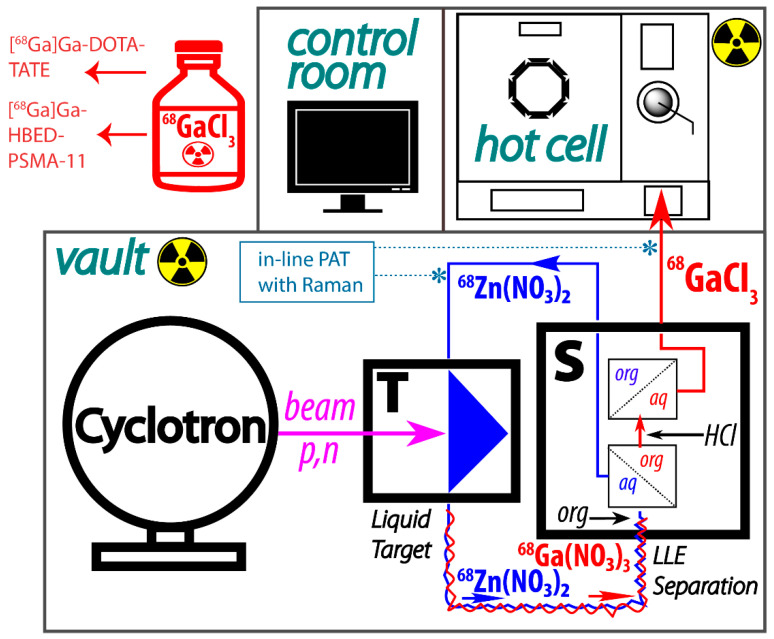
Conceptual schematic of the continuous production of ^68^Ga using a cyclotron solution target (**T**). Phase separation is performed in module **S** using a membrane separator; the ^68^Ga is back-extracted from the organic phase with 0.1 M HCl and is sent directly into the hot cell. The organic phase is discarded.

We have recently reported a proof-of-principle study describing efficient liquid–liquid extraction of radiogallium (^66,67,68^Ga) from ZnCl_2_/HCl solutions in batch and in flow using a membrane-based separator [21]. We argued that, compared to SPE, liquid–liquid extraction in flow (LLEF) has the advantages of scalability, speed, low cost, and easy recovery of the material through solvent evaporation or back-extraction. Importantly, LLEF does not change the chemical composition of the cyclotron liquid target, which can potentially be reused in-line. The ability to reuse the cyclotron target solution of ^68^Zn, coupled with the fluidics-compatible design of solution targets, makes the continuous flow approach an attractive alternative to conventional batch processing. The continuous process can be envisioned as follows: the separation module S is placed next to the liquid target T in the cyclotron vault, and the target is irradiated by the cyclotron beam producing ^68^Ga via the ^68^Zn(*p,n*)^68^Ga nuclear reaction (Figure 1). The irradiated solution is then transferred into S, where ^68^Ga is separated from ^68^Zn. ^68^Ga is sent into the hot cell for further downstream processing and radiolabeling. The cyclotron target solution containing ^68^Zn is returned to T for new irradiation. The process can be performed in a semi-batch mode, where the target is closed during the bombardment and then opened and processed. Alternatively, the cyclotron target solution can be recirculated through **S**.

There are several advantages to the continuous approach: (1) The process is scalable and can be run on-demand. This flexibility translates into the maximization of PET scanner occupancy at the hospital. (2) ^68^Zn is recycled in-line, saving money and securing the hospital’s ^68^Ga and ^68^Zn supply. (3) Radioactive waste remains contained until the completion of the continuous production campaign. (4) Compatibility with in-line process analytical technology tools (PAT) is an opportunity to implement the FDA’s Quality by Design approach to radiopharmaceutical production. In terms of process integration and automatization, LLEF is fully compatible with downstream SPE-based processing, if additional steps are required. Setting the stage for future research, the new liquid extractants can also be used for transferring the solution chemistry to an SPE platform by grafting the extractant onto a solid support.

Central to this conceptual design is a liquid–liquid extraction (LLE)-based separation module. We previously reported near-quantitative LLE of titanium-45 [22,23] and radiogallium [21] from concentrated HCl using a membrane separator with integrated pressure control. The same system could be implemented here with one important caveat: no compound capable of efficient and selective extraction of gallium from nitric acid in the presence of zinc into an organic phase has been reported. Phosphine oxides (Cyanex 923 [24], Cyanex 925 [25]) and phosphoric, phosphonic [26], and phosphinic acids (Cyanex 301 [27]) have been previously tested but extraction was poor.

This study had three objectives. First, we set out to design, synthesize, and test a new family of extractants able to efficiently perform LLE and separation of ^68^Ga from zinc nitrate/nitric acid solutions in batch and in flow. Second, we explored the possibility of implementing Raman spectroscopy as a PAT tool and used the Design of Experiment (DoE) technique for LLE optimization. Lastly, we sought to rationalize the results in the context of the extractant’s structure and its physical properties.

## 2. Results

### 2.1. System Design

**Figure 2:**
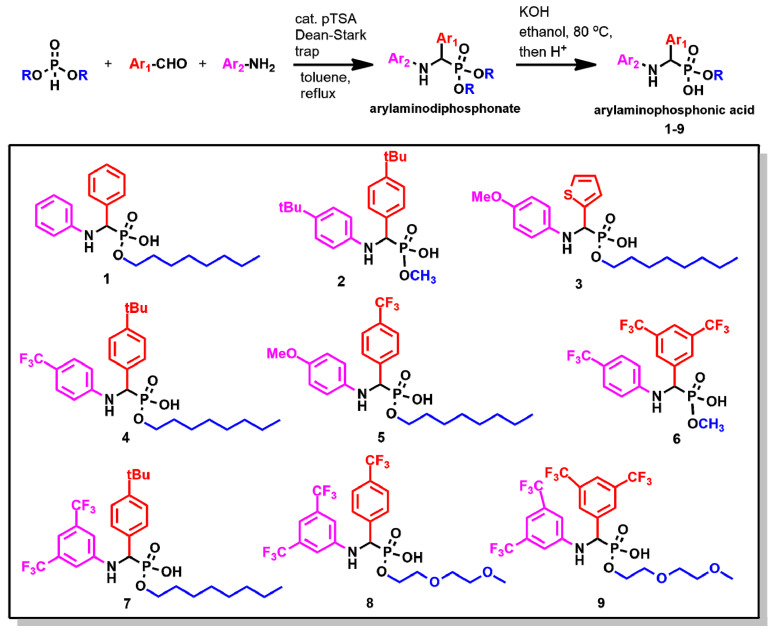
The synthesis of arylamino phosphonic acids **1**–**9**.

The cyclotron target solutions used in clinics can be prepared in a variety of nitric acid concentrations: from 0.01 M to 1.5 M [15,18,28,29]. From an extraction standpoint, the lowest acidity is preferred. Since 1 M solution of ^68^Zn(NO_3_)_2_ in 0.01 M HNO_3_ is also commercially available from Fluidomica for clinical production of ^68^Ga, it became our choice of aqueous phase for LLE. In the 1960s, Jagodić reported that arylamino phosphonic acid **1** was able to extract a broad range of metals from various acids [30]. Therefore, we have chosen the arylamino phosphonic acid scaffold for further development, recognizing that it can function as a chelator due to the presence of arylamino moieties (Figure 2). Importantly, the acidity and basicity of NH, and hence the chelation capacity of the arylamino phosphonic acid extractant, can be controlled by the judicious choice of substituents on the aryl rings Ar_1_ and Ar_2_. Organic and aqueous solubility can be further tuned by controlling the hydrophilicity of the phosphonic acid monoester moiety.

### 2.2. Chemistry

Eight new arylamino phosponic acids **2**–**9**, together with the previously reported acid **1** (Figure 2), were synthesized. Under the conditions of the Kabachnik–Fields reaction [31], a dialkylphosphite, an aniline, and an aldehyde were refluxed in dry toluene in the presence of the catalytic amount of para-toluene sulfonic acid (pTSA) providing the corresponding aminophosphonate as a single product. The subsequent hydrolysis yielded the requested aminophosphonic monoesters (**1**–**9**). The synthesis could be conveniently performed as a one-pot, two-step reaction with overall yields in the 40–50% range.

### 2.3. LLE of ^68^Ga Using Extractants ***1**–**9*** in Batch

The results of LLE of ^68^Ga in batch and the calculated pKa and aqueous solubility of extractants **1**–**9** in different solvents at either room temperature or 50 °C are presented in Table 1.

### 2.4. Estimation of pKa Using COSMO-RS

The pKa for compounds **1**–**9** was computationally estimated using the conductor-like screening model for real solvents (COSMO-RS). This computational technique uses density functional theory to calculate molecular screening charge densities and then applies statistical thermodynamics to yield chemical potentials [32]. pKa can be calculated from the Gibbs free energies of the neutral and ionic compounds [33]. Table 1 shows that the calculated pKa value varies from 1.5 (**1**) to 0.4 (**9**). A significant increase in acidity was noted for all extractants functionalized with the CF_3_ groups (Table 1, entries 6–9).

### 2.5. Batch LLE Optimization Studies

**Figure 3:**
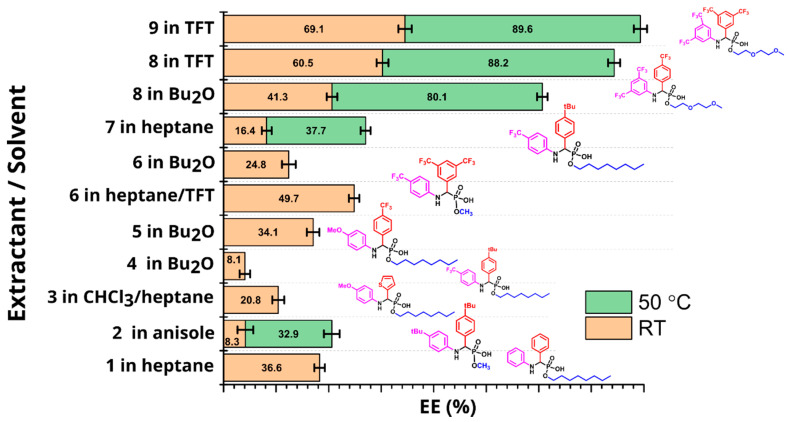
LLE of ^68^Ga using extractants **1**–**9** in batch. In each case, the solvent system was chosen in such a way as to provide the best solubility and phase separation for a given extractant.

Compounds **1**–**9** demonstrated significant variability in aqueous solubility, pKa calculations, and extraction efficiency (EE) (Table 1). The compounds where both Ar_1_ and Ar_2_ were functionalized with trifluoromethyl groups showed the highest EE. A 7–17% increase in extraction was observed when the concentration of the extractants increased from 10 mM to 30 mM (Figure S1). A marked solvent dependence was also noted. For most extractants, the best results were obtained in TFT, heptane, and Bu_2_O. Across all tested compounds, an increase in temperature from RT to 50 °C led to a significant increase in EE (Figure 3). In all cases, clear phase separation between the aqueous and organic phases was observed with no detectable extraction of zinc into the organic phase as evidenced by ^65^Zn activity measurements. Preliminary experiments indicated that, for an extractant in a given solvent, the concentration of the extractant and the extraction temperature were the main factors affecting extraction efficiency. A traditional approach to optimization is to change one factor (concentration, temperature) at a time. This approach, however, is inefficient as it does not necessarily lead to the optimal experimental conditions, especially when there is interaction between the factors. A better approach is to use a statistically-driven experimental design, called the Design of Experiment (DoE) approach [34]. Guided by a software algorithm, DoE allows one to define a design space and systematically optimize the response (EE) while taking into account the interaction between the factors. To optimize the yield of LLE, we used a central composite face-centered design algorithm implemented in the software package MODDE 9.1.1 (Table 2). Concentration and temperature were varied between 10–30 mM and 25–50 °C, correspondingly. These limits defined the center point (20 mM, 37.5 °C) around which four corner and four median experiments were constructed (see Figure S2 for a graphical representation of the design). The reproducibility of the design was estimated by running the center points in triplicate.

**Figure 4:**
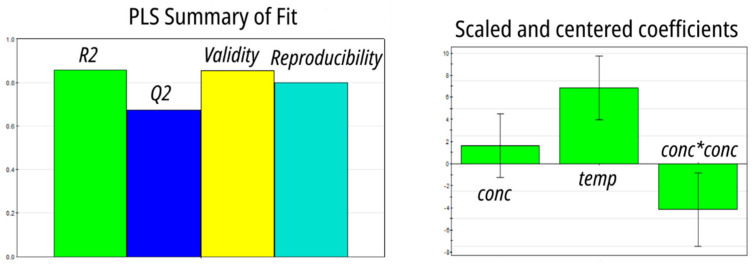
Optimization of LLE in batch using extractant **8**: a PLS model based on a central composite face-centered design.

The results of 11 runs were fitted with the partial least squares (PLS) algorithm, producing a quadratic model of excellent statistical quality (Figure 4). Both concentration and temperature were positively correlated with LLE efficiency, but the square of concentration was negatively correlated.

The optimization studies indicated that the extractions performed at and above 20 mM and 37.5 °C consistently produced EE > 83% (Table 2).

### 2.6. LLE of ^68^Ga using Extractants ***8*** and ***9*** in Continuous Flow

**Figure 5:**
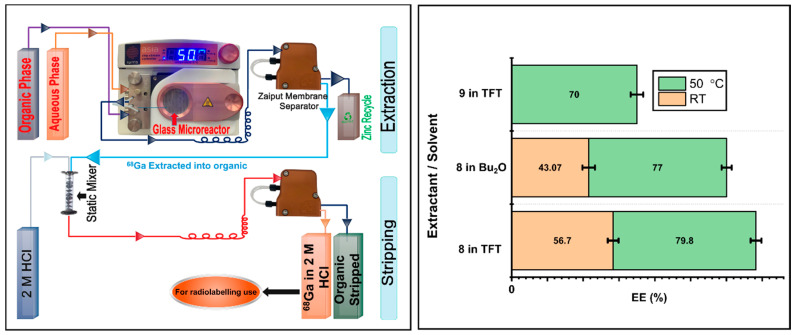
(**Left**) A schematic depicting two-stage liquid–liquid extraction in flow. (**Right**) ^68^Ga EE (%) obtained from a 1 M Zn(NO_3_)_2_ solution in 0.01 M HNO_3_ using extractants **8** and **9** in TFT and Bu_2_O at RT and 50 °C.

Having established extractants **8** and **9** as the best performers in batch, we translated batch into flow (Figure 5). The Zaiput membrane separator provided a clean phase separation with no phase breakthrough at both the extraction and stripping stages. At the extraction stage, LLE was on average 10% less efficient in flow. As with the batch experiments, raising the temperature to 50 °C significantly improved EE. Stripping in 2 M HCl was quantitative. ICP analysis of the stripped solution indicated the presence of 11 ppm of zinc.

### 2.7. Zinc Nitrate Quantification Using Raman Spectra

**Figure 6:**
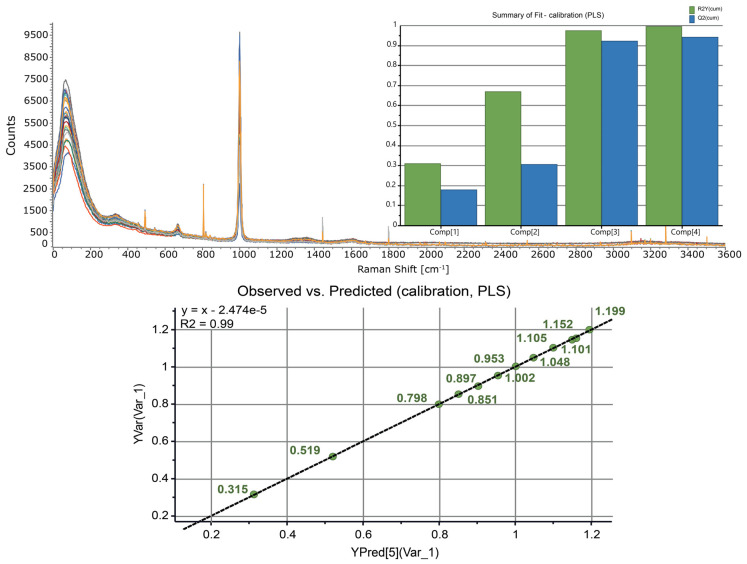
(**Top**) The Raman spectra of Zn(NO_3_)_2_ (0.3−1.2 M) prepared in 0.01 M HNO_3_ solutions. (**Inset**) A four-component PLS calibration model for determination of zinc concentration. (**Bottom**) The performance of the calibration model: observed vs. predicted.

Twelve solutions of zinc nitrate in 0.01 M HNO_3_ were prepared (with the concentration of zinc varying in the range of 0.3–1.2 M) and the Raman spectra were acquired. A multivariate analysis of the spectra using SIMCA yielded a four-principal component PLS model with R^2^ = 0.99 and Q^2^ = 0.94 (Figure 6). The model showed excellent observed vs. predicted linearity (R^2^ = 0.99) in the whole concentration range and was validated using independently prepared samples. This multivariate calibration was subsequently used to quantify the concentration of zinc before and after LLE.

The analysis showed that the overall depletion of zinc in the aqueous phase after LLE was less than 10%. This was independently confirmed by the mass balance measurement and ^65^Zn radiotracing.

## 3. Discussion

The substituents on Ar_1_, Ar_2_, and the phosphonic acid affect the properties of compounds **1**–**9** in two major ways. The electron-withdrawing CF_3_ groups increase the acidity of the phosphonic acid moiety, whereas electron-releasing tBu and OMe retard dissociation. This is reflected in the calculated pKa value, which varies by more than 1 pKa unit across the series. Experimentally, we found that the increase in the strengths of the phosphonic acid moiety due to CF_3_ substitution resulted in compounds **8** and **9** being stronger extractants. This is in line with the general observation that the potassium salts of compounds **1**–**9** proved to be better extractants than the corresponding acids. The effect was particularly pronounced in **4**: EE increased from 8% for the phosphonic acid to 75% for its potassium salt. A similar effect was previously observed by Jagodić and attributed to an increase in the acidity of the solution due to the release of H^+^ during complexation [30]. Although this explanation is not applicable to the present case due to the picomolar amount of ^68^Ga, the importance of phosphonic acid dissociation is further highlighted by the critical role aqueous phase acidity played in extraction. When **1** was used as an extractant, EE dropped from 30% in 0.01 M HNO_3_ to 4.2% in 0.2 M HNO_3_, and then to 1.2% in 1 M HNO_3_. Despite a qualitative correlation between pKa and EE, no statistically significant model emerged from the data. The effect of substituents in Ar_1_ and Ar_2_ on the solubility of compounds **1**–**9** is even more subtle, as both electronic and steric effects play a role. The substitution of an octyl group for a hydrophilic diethylene glycol significantly increased the aqueous solubility of **8** and **9**. Although no model was able to correlate solubility and EE, the combination of COSMO-RS-calculated acidity and solubility yielded a statistically significant PLS model (Figure S3). The combination of higher acidity and higher aqueous solubility favored extraction. A marked solvent, temperature, and concentration dependency can be further rationalized in terms of extractant dimerization, which in the case of alkyl and aryl phosphonic acids has been observed in solvents of low polarity [35]. The negative correlation between EE and the square of the concentration of the extractant we found during batch optimization (Figure 4) suggests that dimerization of extractant **8** competes with extraction. Under this scenario, the rate of dimerization would be proportional to the square of the concentration and would lead to a decrease in EE due to the deactivation of the extractant. Increased temperature is expected to favor the dissociation of the dimer, leading to higher EE. The higher EE of the potassium salts of **1**–**9** we observed in the preliminary experiments also supports the dimerization hypothesis because the deprotonated species are unable to form aggregates.

Aqueous solutions are essentially transparent to Raman scattering. In our continuous flow design, the laser light was delivered to the flow cell via fiber optics, making Raman spectroscopy an ideal tool for remotely controlled in-line analysis of radioactive mixtures under continuous flow conditions. The Raman spectra of 0.3–1.2 M Zn(NO_3_)_2_ prepared in 0.01 M HNO_3_ solutions were dominated by the symmetric stretching band of nitrate anion centered at 1000 cm^−1^ (Figure 6). The broad peak spanning 305–399 cm^−1^ was assigned to the hexaaquazinc(II) ion [Zn(H_2_O)_6_]^2+^ (390 cm^−1^) [36] and the lower-frequency mode corresponding to the nitrate-associated [Zn(H_2_O)_x_NO_3_]^+^, x < 6 [37]. We found that inclusion of the entire spectral region (3–3600 cm^−1^) and autoscaling the variables to unit variance were essential for obtaining calibrations with the best possible statistical quality.

## 4. Materials and Methods

### 4.1. Materials

All chemicals were reagent grade, purchased from Sigma Aldrich (Merck KGaA, Darmstadt, Germany), and used without additional purification. Dioctyl and diethyleneglycol phosphites were prepared as described previously [38]. The batch and continuous flow extractions were performed using zinc nitrate at natural abundance, spiked with a small amount of zinc-65 for radiotracing purposes. The radionuclide zinc-65 (^65^Zn, t½: 244 days) was produced as described previously [21]. The radionuclide gallium-68 (^68^Ga, t½: 68 min) was obtained from a ^68^Ge/^68^Ga generator produced by Eckert & Ziegler (Berlin, Germany). SEP-10 membrane separators were purchased from Zaiput Flow Technologies (Waltham, MA, USA). Pall PTFE membranes were used for all experiments (47 mm diameter, 0.2 µm pore size, polypropylene support). Perfluoroalkoxy alkane (PFA) diaphragms (0.002″ (00.0508 mm)) were purchased from McMaster Carr (Princeton, NJ, USA). All PFA tubing (1/16″ (1.5875 mm) OD, 0.03″ (0.762 mm) ID) was purchased from Idex Health and Science (West Henrietta, NY, USA). Polytetrafluoroethylene (PTFE) static mixers were purchased from Stamixco (Dinhard, Switzerland). The 15 mL plastic centrifuge tubes with screw caps (SuperClear) were purchased from VWR (Søborg, Denmark).

### 4.2. Instrumentation and Methods

The NMR spectra were recorded on an Agilent 400 MR spectrometer (Agilent, Santa Clara, CA, USA) operating at 400.445 MHz (1H). The Raman spectra were obtained using an Avantes AvaSpec-ULS-RS-TEC spectrometer with 788 nm laser excitation. Zinc-65 and gallium-68 were quantified by gamma spectroscopy using a Princeton Gammatech LGC 5 or Ortec GMX 35195-P germanium detector, calibrated using certified barium-133 and europium-152 sources. Zinc at natural abundance was quantified using a Thermo Scientific iCAP 6000 Series ICP Optical Emission Spectrometer. An Eppendorf 5702 centrifuge was used to assist in phase separation. All experiments used 0.2 µm membrane pore size, a 0.002” (0.051 mm) diaphragm, two 10-element static mixers, and a 108 cm mixing tube. The solutions for the continuous membrane-based separation were pumped using KDS 100 Legacy Syringe pumps. For batch experiments, phase mixing was performed using an IKA ROCKER 3D digital shaker.

### 4.3. Batch LLE Extractions

Initially, 1 M zinc nitrate prepared in 0.01 M HNO_3_ was used as the aqueous phase for the batch LLE extraction. The organic phase was prepared by dissolving extractants **1**–**9** in different organic solvents or a mixture of solvents to achieve a final concentration of 10 mM, 20 mM, and 30 mM. A 1 mL aliquot of the aqueous phase was then transferred into a 15 mL centrifuge tube and mixed with 3 mL of the organic phase. The tube was shaken at 80 rpm for 15 min at either 25 °C, 37.5 °C, or 50 °C. After centrifugation for 15 min, the phases where separated and the activity of ^65^Zn and ^68^Ga in the aqueous and organic phases were quantified using gamma spectroscopy. ^68^Ga extraction efficiency was calculated according to the following equation:

EE(%) = (^68^Ga_org_ × 100%)/(^68^Ga_org_ + ^68^Ga_aq_)

### 4.4. Continuous Membrane-Based LLE

LLE in flow was performed in two stages. At first, the extraction stage used the same aqueous phase as was used in batch LLE. Extractants **8** and **9**, which showed the best performance in batch extraction, were used as 10 mM TFT solutions for the organic phase. The extraction was performed at 50 °C. The fluidics were driven by two separate syringe pumps equipped with Hamilton glass syringes, which were filled with the organic (9 mL) and the aqueous (3 mL) phases. The aqueous flow rate was 15 mL/h and the organic flow rate was 45 mL/h for all experiments. The aqueous to organic ratio was maintained at 1:3 (*v*/*v*) at all times. For the extraction process, two phases passed through PFA tubing (1/16’’ OD, 0.03’’ ID) and entered the Syriss Chip Climate controller mixer set at 50 °C. The organic and aqueous phases were mixed inside the chip. The two phases were further mixed in a 100 cm long PFA tubing (1/16’’ OD, 0.03’’ ID) mixing loop by steady slug flow and then passed into the membrane separator. In the membrane separator, the organic phase permeated the hydrophobic membrane (PTFE/PP, 0.2 µm pore size and 139 µm thickness) and passed through the permeate outlet, while the aqueous phase was retained and passed through the retentate outlet. The 0.002” PFA diaphragm in the membrane separator worked as a form of integrated pressure control, and complete phase separation between the aqueous and the organic phase was obtained with the chosen membrane and diaphragm. In the second stage (stripping), ^68^Ga was back-extracted from the organic phase into the aqueous phase following the experimental protocol described above. This time, however, the organic and aqueous phases were mixed by two PTFE static mixers at room temperature. Again, the aqueous to organic ratio was maintained at 1:3 (*v*/*v*), where 9 mL of organic phase containing ^68^Ga was stripped with 3 mL of 2 M HCl.

### 4.5. NMR Studies

#### 4.5.1. General Comments

NMR spectra were recorded at ambient probe temperatures and referenced as follows (δ, ppm): ^1^H, residual internal DMSO-d5 (2.50); ^13^C{^1^H} internal DMSO-d6 (39.52). The partial structural assignment was performed on a basis of one-dimensional nuclear Overhauser effect spectroscopy (1D NOESY), Heteronuclear Multiple Bond Correlation with adiabatic pulses (HSQCAD), and Gradient-Selected Correlation Spectroscopy (gCOSY) experiments. qNMR studies were performed at ambient temperature using the following acquisition parameters: ^1^H NMR—wet1D pulse sequence with 90^o^ pulse, acquisition time 4 s, relaxation delay 60 s, zero-filling to 256 k, exponential multiplication with 0.3 Hz line broadening, manual phasing, and 5th degree polynomial baseline correction. ^31^P NMR—inverse gated decoupling pulse sequence with 90° pulse, acquisition time 1 s, relaxation delay 30 s, zero-filling to 128 k, exponential multiplication with 3 Hz line broadening, manual phasing, and 5th degree polynomial baseline correction.

#### 4.5.2. Determination of the Aqueous Solubility of Extractants **1**–**9**

A 1 mL volumetric flask was charged with 10–15 mg of the extractant and 1 mL of D_2_O. The resulting suspension was sonicated for 30 min and the content was centrifuged, filtered through a 0.45 µm syringe filter, and transferred into an NMR tube. A sealed capillary containing an external calibrant (acetanilide for ^1^H qNMR and triphenylphosphine for ^31^P qNMR) was inserted into the NMR tube and the solubility of extractants **1**–**9** was determined from their ^1^H or ^31^P NMR spectra using the formula: C_x_ = (I_x_/I_cal_) × (N_cal_/N_x_) × C_cal_, where I, N, and C are the integral area, number of nuclei, and the concentration of the extractant (x) and the calibrant (cal), respectively.

### 4.6. Multivariate Analysis

The Design of Experiment (DoE) studies were performed using MODDE 9.1.1 (Umetrics AB), using the central composite face-centered design. Multivariate calibration of the zinc nitrate concentrations was performed by acquiring the corresponding Raman spectra using 788 nm laser excitation with 5 s acquisition (10 average) and exporting the spectra as GRAMS SPC files into SIMCA 17.0.0 (Sartorius Stedim Data Analytics AB). The dataset (3–3600 cm^−1^) was scaled to unit variance and processed using PLS. Five independently prepared samples were used for validation of the calibration model.

### 4.7. Computational Methods

All gas-phase and COSMO calculations were performed using the TURBOMOLE 7.5.1 suite of programs using resolution of identity approximation (RI) [39]. The gas-phase structures were optimized at the RI BP/def2-TZVPD level and convergence to the ground state was verified by running analytical frequency calculations. The single-point gas-phase energies were then re-evaluated at the RI MP2/def2-TZVPP level. The COSMO files were obtained at the same theory level in the COSMO phase with a smooth radii-based isosurface cavity, and convergence to the ground state was verified by running numerical frequency calculations. The resulting COSMO files were used to perform solution thermodynamics calculations using COSMOtherm, Version 20.0.0 (Dassault Systèmes), yielding the free energies of solvation, sigma surfaces, and sigma profiles [40].

### 4.8. General Procedure for the Synthesis of Arylaminophosphonic Acids ***1**–**9***

A 100 mL round-bottom flask equipped with the Dean–Stark trap was charged with an equimolar (5 mmol) amount of a dialkylphosphite, an aniline, an aldehyde, and a catalytic amount of pTSA dissolved in 40 mL of toluene. The reaction mixture was refluxed overnight. Toluene was removed under reduced pressure, the reaction mixture was redissolved in 50 mL of ethanol, and a solution of 10 mmol of KOH in 4 mL of water was added. The reaction mixture was refluxed overnight, cooled to room temperature, acidified with 3 eq (15 mmol) of 6 M HCl, and purified on silica (gradient elution: CHCl_3_ then CHCl_3_/CH_3_OH 9/1 *v*/*v*). Analytical purity was confirmed by qNMR as described above.

## 5. Conclusions

A new family of arylaminophosphonic acids (**1**–**9**) was synthesized. All compounds selectively extracted gallium-68 in the presence of zinc from a solution containing 1 M Zn(NO_3_)_2_ in 0.01 M HNO_3_. Extractants **8** and **9** were able to extract up to 90% of gallium in batch and up to 80% in flow. Only ppm amounts of zinc were co-extracted. Extraction efficiency correlated with pKa and the aqueous solubility of the extractant. Raman spectroscopy was found to be well suited as a PAT tool for continuous flow production of gallium-68. We are currently evaluating the suitability of cyclotron-produced and LLEF-purified gallium-68 for DOTATATE radiolabeling.

## Figures and Tables

**Table 1:** Batch LLE of ^68^Ga from 1 M zinc nitrate in 0.01 M nitric acid with 10 mM of extractant dissolved in various solvents and performed at RT or 50 °C. The extraction efficiencies (EE), the pKa of the extractants calculated using Cosmotherm, and the experimentally determined aqueous solubilities are listed. Experiments were performed in triplicate; results are presented as means ± standard deviation.

Entry Extractant	Solvent	T, °C	^68^Ga EE (%)	pKa^Calc^	Aqueous Solubility, mM
1	Heptane	RT	30.6 ± 2.0	1.5	0.22
2	Anisole	RT	8.3 ± 3.1	1.4	0.10
		50 °C	32.9 ± 2.3		
3	CHCl_3_/Heptane 3/1 (*v*/*v*)	RT	20.8 ± 2.0	1.4	0.25
4	Bu_2_O	RT	8.1 ± 2.4	1.2	0.08
5	Bu_2_O	RT	34.1 ± 2.0	1.2	0.04
6	Heptane/TFT1:1 (*v*/*v*)	RT	49.7 ± 2.6	0.7	2.60
	Bu_2_O	RT	24.8 ± 1.9		
7	Heptane	RT	16.4 ± 2.0	0.9	0.08
		50 °C	37.7 ± 2.3		
8	Bu_2_O	RT	41.3 ± 2.5	0.7	1.60
		50 °C	80.1 ± 3.0		
	TFT	RT	60.5 ± 1.9		
9	TFT	50 °C	88.2 ± 2.3		
		RT	69.1 ± 3.0	0.4	1.73
		50 °C	89.6 ± 3.5		

**Table 2:** Batch LLE optimization studies using the central composite face-centered design algorithm implemented in MODDE 9.1.1. Concentration and the temperature were simultaneously varied.

Exp. No.	Concentration, mM	T, °C	EE (%)
1	10	25	69
2	30	25	71
3	10	50	83
4	30	50	91
5	10	37.5	75
6	30	37.5	78
7	20	25	71
8	20	50	90
9	20	37.5	83
10	20	37.5	88
11	20	37.5	91

## Data Availability

Not applicable.

## Supplementary Materials

The following supporting information can be downloaded at: https://www.mdpi.com/article/10.3390/molecules27238377/s1. Figure S1: The extraction efficiencies for compound **8**, performed at 10 mM and 30 mM in various solvents at RT and 50 °C. The relative increase in EE upon the increase in concentration of compound **8** from 10 to 30 mM is shown on the X axis. Table S1: The distribution ratio Ds = [^68^Ga_tot_]org/[^68^Ga_tot_]_aq_ for batch LLE of ^68^Ga from 1 M zinc nitrate in 0.01 M nitric acid with 10 mM of extractant dissolved in various solvents and performed at RT or 50 °C. Figure S2: The design region for the central composite face-centered design shown in Table S1. The numbers in the circles correspond to the experiment numbers in Table S1. The center point was run in triplicate (experiments 9, 10, 11). Figure S3: A PLS model correlating ^68^Ga extraction efficiency with pKa and the aqueous solubility of compounds **1**–**9**.

## Sample Availability

Samples of the compounds are not available from the authors.
