# Supplementary material for: Recovery of Gallium-68 and Zinc from HNO3-Based Solution by Liquid–Liquid Extraction with Arylamino Phosphonates
Source: Molecules. 2022 Dec 1;27(23):8377. doi: 10.3390/molecules27238377 (PMC9735965; doi:10.3390/molecules27238377)
Supplement: Supplementary file 1 [file molecules-27-08377-s001.zip › molecules-2014434-supplementary.pdf]

Supplementary Materials: Recovery of Gallium-68 and Zinc from HNO_3-Based Solution by Liquid–Liquid Extraction with Arylamino Phosphonates

Fedor Zhuravlev [1,*], Arif Gulzar [1] and Lise Falborg [2]

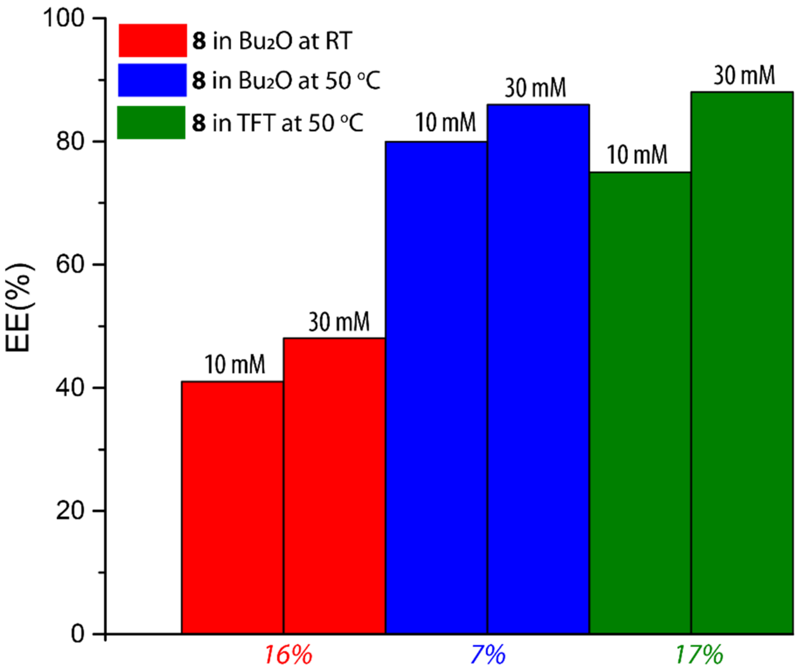

Figure S1. The extraction efficiencies for compound 8, performed at 10 mM and 30 mM in various solvents at RT and 50 °C. The relative increase in EE upon the increase in concentration of 8 from 10 to 30 mM is shown on the X axis.

Table S1. The distribution ratio Ds=$[^{68}Ga_{tot}]_{org}/[^{68}Ga_{tot}]_{aq}$ for batch LLE of ^{68}Ga from 1 M zinc nitrate in 0.01 M nitric acid with 10 mM of extractant dissolved in various solvents and performed at RT or 50 ^oC.

Entry	Extractant	Solvent	T, oC	Ds
1	1	Heptane	RT	10.2
2	2	Anisole	RT	2.8
3	2	Anisole	50 ^oC	11.0
4	3	CHCl3/Heptane, 3/1 (v/v)	RT	7.0
5	4	Bu_2O	RT	2.7
6	5	Bu_2O	RT	11.4
7	6	Heptane/TFT1:1(V/V)	RT	16.6
8	6	Bu_2O	RT	8.3
9	7	Heptane	RT	5.5
10	7	Heptane	50 ^oC	12.6
11	8	Bu_2O	RT	13.8
12	8	Bu_2O	50 ^oC	26.7
13	8	TFT	RT	20.2
14	8	TFT	50 ^oC	29.4
15	9	Bu_2O	RT	23.0
16	9	Bu_2O	50 ^oC	29.9

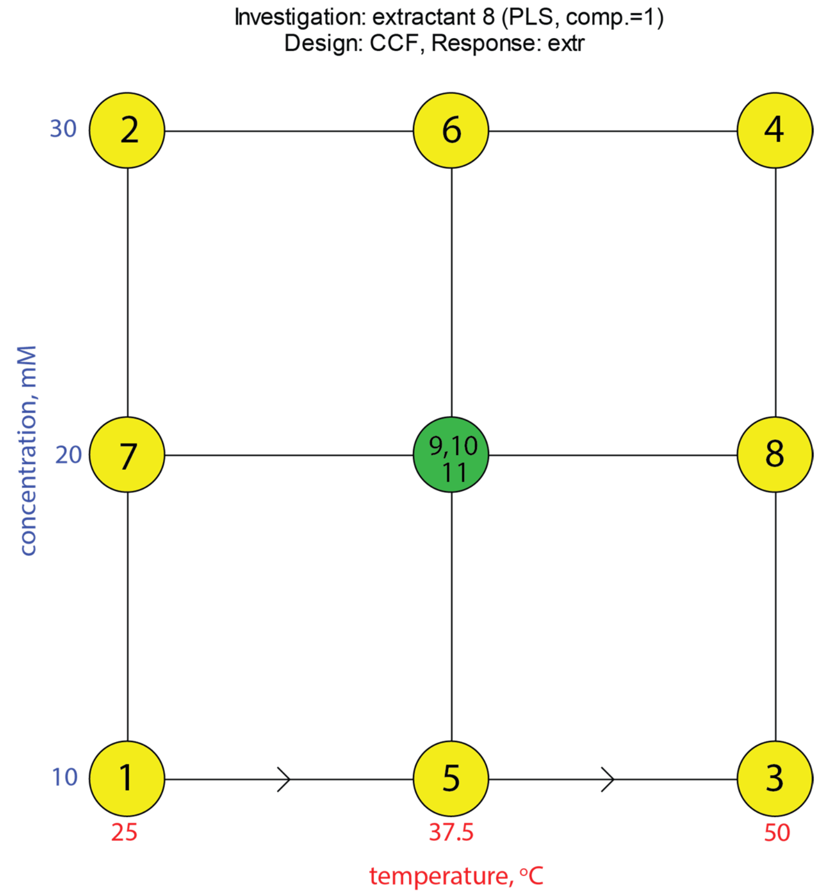

Figure S2. The design region for the central composite face-centered design shown in Table S1. The numbers in the circles correspond to the experiment numbers in Table S1. The center point was run in triplicate (experiments 9,10,11).

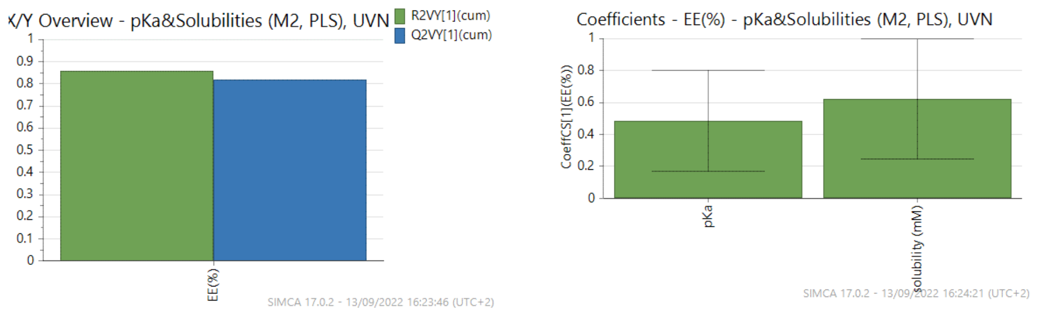

Figure S3. A PLS model correlating the ^{68}Ga extraction efficiency with pKa and aqueous solubility of compounds 1-9

Spectroscopic data for arylaminophosphonic acids 1-9

1 was obtained using the general procedure in 45% yield

NMR (dmso-*d6*, δ in ppm): ^{1}H (400.25 MHz) 7.52–7.45 (apparent d, 2H), 7.32–7.23 (apparent t, 2H), 7.22–7.15 (apparent t, 1H), 7.01–6.94 (apparent t, 2H), 6.72–6.66 (apparent d, 2H), 6.52–6.46 (apparent t, 1H), 4.77 (d, J = 24.5 Hz, 1H), 3.89–3.68 (m, 2H), 1.49–1.37 (m, 2H), 1.31–1.11 (m, 10H), 0.85 (t, J = 6.8 Hz, 3H). $^{13}C\{^{1}H\}$ (101 MHz) δ, 147.48 (d, J = 14.1 Hz), 138.23, 128.57, 128.20 (d, J = 5.3 Hz), 127.73 (d, J = 2.7 Hz), 126.77 (d, J = 3.1 Hz), 116.4, 113.25, 65.39 (d, J = 6.9 Hz), 54.82 (d, J = 147.6 Hz), 31.18, 30.10 (d, J=5.6 Hz), 28.59, 28.55, 25.01, 22.06, 13.94. $^{31}P\{^{1}H\}$ (162 MHz) 19.19.

2 was obtained using the general procedure in 44% yield.

NMR (dmso-*d6*, δ in ppm): ^{1}H (400.25 MHz) 7.44-7.36 (m, 2H), 7.34-7.27 (m, 2H), 7.05-6.97 (apparent d, 2H), 6.69-6.61 (apparent d, 2H), 4.76 (d, J = 24.9 Hz, 1H), 3.53 (d, J = 10.6 Hz, 3H), 1.25 (*s*, 9H), 1.16 (*s*, 9H). $^{13}C\{^{1}H\}$ (101 MHz) δ , 149.18 (d, J = 3.1 Hz), 144.93 (d, J = 14.9 Hz),

138.75, 134.85 (d, J = 1.1 Hz), 127.86 (d, J = 5.3 Hz), 125.27, 124.67 (d, J = 2.5 Hz), 112.94, 54.03 (d, J = 149.7 Hz), 52.49 (d, J = 6.3 Hz), 34.15 (d, J = 1.1 Hz), 33.41, 31.38, 31.16. $^{31}P\{^1H\}$ (162 MHz) 23.48.

3 was obtained using the general procedure in 51% yield.

NMR (dmso-*d6*, δ in ppm): ^1H (400.25 MHz) 7.37-7.31 (apparent d, 1H), 7.16–7.10 (m, 1H), 6.96-6.91 (m, 1H), 6.75-6.69 (m, 2H), 6.67–6.61 (m, 2H), 5.00 (d, J = 24.1 Hz, 1H), 3.94–3.76 (m, 2H), 3.60 (s, 3H) 1.52–1.41 (m, 2H), 1.30–1.10 (m, 10H), 0.84 (t, J = 6.9, 3H). $^{13}C\{^1H\}$ (101 MHz) δ , 151.49, 141.99, 141.16 (*d*, J = 13.9 Hz), 126.44 (*d*, J = 2.3 Hz), 126.02 (*d*, J = 7.0 Hz), 124.83 (*d*, J = 3.2 Hz), 114.58, 114.23, 65.70, 65.63, 51.72 (*d*, J = 155.1 Hz), 31.19, 30.08 (*d*, J = 6.1 Hz), 28.59, 28.54, 24.98, 22.05, 13.9. $^{31}P\{^1H\}$ (162 MHz) 18.37.

4 was obtained using the general procedure in 41% yield.

NMR (dmso-*d6*, δ in ppm): ^1H (400.25 MHz) 7.47-7.39 (m, 2H), 7.36–7.26 (apparent t, 4H), 7.03-6.93 (m, 1H), 6.90-6.84 (apparent d, 2H), 4.95-4.80 (apparent dd, 1H), 3.92–3.72 (m, 2H), 1.53-1.36 (m, 2H), 1.35-1.03 (m, 19H), 0.83 (t, J = 7.0 Hz, 3H). $^{13}C\{^1H\}$ (101 MHz) δ , 150.80 (d, J = 13.5 Hz), 149.39 (d, J = 3.1 Hz), 141.61 (d, J = 13.9 Hz), 134.21, 127.91 (d, J = 5.3 Hz), 125.9 (d, J = 4.1 Hz), 125.7 (q, J = 270.8 Hz), 124.7 (d, J = 2.2 Hz), 116.09 (q, J=32.1 Hz), 115.91, 112.55, 65.50 (d, J = 6.3 Hz), 53.70 (d, J = 149.9), 34.16, 31.14, 30.04 (d, J = 6.3 Hz), 28.57 (d, J = 4.4 Hz), 25.00, 22.03, 13.89. $^{31}P\{^1H\}$ (162 MHz) 19.28. ^{19}F (377 MHz) -59.11.

5 was obtained using the general procedure in 53% yield.

NMR (dmso-*d6*, δ in ppm): ^1H (400.25 MHz) 7.73-7.61 (m, 4H), 6.71–6.58 (m, 4H), 4.91 (d, J = 25.0, 1H), 3.94–3.75 (m, 2H), 3.57 (*s*, 3H), 1.51-1.38 (m, 2H), 1.29-1.12 (m, 10H), 0.84 (*t*, J = 6.9 Hz, 3H). $^{13}C\{^1H\}$ (101 MHz) δ ,151.33, 143.45, 141.02 (d, J = 15.3 Hz), 128.98 (d, J = 4.5 Hz), 127.48 (q, J = 31.2 Hz), 125.02 (d, J = 6.5 Hz), 124.57, 124.39 (*q*, J = 275.0 Hz), 114.51, 144.26, 65.52 (d, J = 6.4 Hz), 55.22 (d, J = 148.0 Hz), 55.12, 31.17, 30.03 (d, J = 5.6 Hz), 28.56 (d, J = 5.8 Hz), 24.97, 22.04, 13.89. $^{31}P\{^1H\}$ (162 MHz) 19.10. ^{19}F (377 MHz) -60.83.

6 was obtained using the general procedure in 50% yield.

NMR (dmso-*d6*, δ in ppm): ^1H (400.25 MHz) 8.25 (apparent s, 2H), 8.00 (apparent s, 1H), 7.37-7-24 (m, 3H), 7.0-6.91 (apparent d, 2H) 5.53-5.39 (apparent dd, 1H), 3.59 (d, J=10.5 Hz, 3H).). $^{13}C\{^1H\}$ (101 MHz) δ , 150.30 (d, J = 12.3 Hz), 141.59, 129.85 (q, J = 32.8), 128.9 (br s), 126.56 (d, J = 4.0 Hz), 125.07 (q, J = 269.8 Hz), 123.40 (q, J = 273.2 Hz), 121.07 (br s), 116.84 (q, J = 31.9 Hz), 112.75, 52.81 (d, J = 147.1 Hz), 53.06 (d, J = 6.5 Hz). $^{31}P\{^1H\}$ (162 MHz) 18.64. ^{19}F (377 MHz) -59.25 (s, 3F), -61.21 (s, 6F).

7 was obtained using the general procedure in 48% yield

NMR (dmso-*d6*, δ in ppm): ^1H (400.25 MHz) 7.49-7.44 (m, 2H), 7.41 (br s, 2H), 7.37-7.30 (apparent d, 3H), 7.02 (br s, 1H), 5.19-5.07 (apparent dd, 1H), 3.94-3.75 (m, 2H), 1.41-1.36 (m, 2H), 1.34-1.05 (m, 19H), 0.88-0.78 (m, 3H). $^{13}C\{^1H\}$ (101 MHz) δ , 149.56 (d, J=3.0 Hz), 149.19 (d, J = 12.0 Hz), 133.7 (J = 0.8 Hz), 130.68 (q, J = 31.9 Hz), 127.97 (d, J = 5.3 Hz), 124.7 (d, J = 2.1 Hz), 123.7 (q, J = 272.9 Hz), 113.05, 108.37, 65.46 (d, J = 6.6 Hz), 53.32 (d, J=149.9 Hz), 34.18, 31.14, 31.10, 30.05, 29.99, 28.55 (d, J = 2.1 Hz), 25.03, 22.00, 13.84. $^{31}P\{^1H\}$ (162 MHz) 19.34. ^{19}F (377 MHz) -61.78.

8 was obtained using the general procedure in 46% yield

NMR (dmso-*d6*, δ in ppm): ^1H (400.25 MHz), 8.25 (br s, 2H), 7.99 (br s, 1H), 7.39-7.32 (apparent d, 2H), 7.29-7.19 (m, 1H), 6.99-6.91 (apparent d, 2H), 5.50-5.36 (apparent dd, 1H), 4.11-3.96 (m, 2H), 3.54-3.40 (m, 4H), 3.39-3.32 (m, 2H), 3.19 (s, 3H). $^{13}C\{^1H\}$ (101 MHz) δ , 150.33 (d, J=13.3 Hz), 147.7, 129.89 (q, J=32.15 Hz), 128.94 (br s), 126.15 (q, J=269.8), 126.11 (d, J=3.1 Hz), 123.44 (q, J= 273.62 Hz), 121.10, 116.87 (q, J=31.9 Hz), 112.79, 71.16, 69.71 (d, J=5.1 Hz), 69.52, 65.18 (d, J=6.3 Hz), 58.01, 53.21 (d, J=147.9 Hz). $^{31}P\{^1H\}$ (162 MHz) 17.75. ^{19}F (377 MHz) -59.26 (s, 3F), -61.20 (s, 6F).

9 was obtained using the general procedure in 41% yield

NMR (dmso-*d6*, δ in ppm): ^1H (400.25 MHz), 8.20 (br s, 2H), 7.88 (br s, 1H), 7.28 (br s, 2H), 6.97 (br s, 1H), 5.28-4.99 (apparent d, 1H), 4.04-3.80 (m, 2H), 3.54-3.31 (m, 8H), 3.17 (s, 3H).). $^{13}C\{^1H\}$ (101 MHz) δ , 149.26 (d, J=13.1 Hz), 143.81, 130.60 (q, J = 32.1Hz), 129.43 (q, J = 32.4Hz), 128.70 (br s), 123.58 (q, J = 272.5Hz), 123.51 (q, J = 273.5Hz), 120.00 (br s), 112.24, 107.61, 71.14, 70.23 (d, J = 4.7 Hz), 69.39, 64.00 (d, J = 5.6 Hz), 57.90, 54.52 (d, J = 138 Hz). $^{31}P\{^1H\}$ (162 MHz) 13.46. ^{19}F (377 MHz) -59.26 (s, 3F), -61.96 (s, 6F), -61.31 (s, 6F).
